# Academic Self-Efficacy and Postgraduate Procrastination: A Moderated Mediation Model

**DOI:** 10.3389/fpsyg.2020.01752

**Published:** 2020-07-24

**Authors:** Guoqing Liu, Gang Cheng, Juan Hu, Yun Pan, Shouying Zhao

**Affiliations:** ^1^School of Psychology, Guizhou Normal University, Guiyang, China; ^2^National Demonstration Center for Experimental and Comprehensive Education, Guizhou Normal University, Guiyang, China; ^3^School of Foreign Languages, Guizhou Normal University, Guiyang, China

**Keywords:** procrastination, graduate students, academic self-efficacy, self-control, gender

## Abstract

Studies in recent years have shown that academic procrastination in postgraduates is very common and has a negative impact on their mental health. Therefore, we conducted this study to explore the influencing mechanism of postgraduate academic procrastination. In this study, based on the Temporal Decision Model (TDM) of procrastination and the strength model of self-control, we administered a questionnaire survey to 577 full-time postgraduates (351 females, 226 males) to explore the influence mechanisms and gender differences of motivational and volitional factors on academic procrastination. Our results indicated significant differences in academic self-efficacy between females and males. Academic self-efficacy was positively correlated with academic self-control and negatively correlated with academic procrastination; academic self-control was negatively correlated with academic procrastination. Academic self-control had a completely mediating effect in the influence of academic self-efficacy on academic procrastination. Gender variables moderated the influence of academic self-efficacy on academic self-control and thus significantly moderated the mediating effect of academic self-control. Specifically, academic self-control had a stronger mediating effect between academic self-efficacy and academic procrastination for female postgraduates. Our findings may provide guidance for postgraduates who exhibit academic procrastination and extend the theory of academic procrastination.

## Introduction

Procrastination means that, in spite of their knowledge of negative effects, people choose to delay their schedules (Sirois et al., 2003). Because individuals know that procrastination can lead to negative consequences and still choose to delay, procrastination has negative effects on emotions, academic performance (Stead et al., 2010), social achievements, subjective well-being (Gueorguieva, 2011), sleep quality (Przepiorka et al., 2019), and even physical health (Klingsieck, 2013). Academic procrastination is a specific kind of procrastination, the manifestation of procrastination in learning (Zhang et al., 2010). Academic procrastination is commonly seen among middle-school, high-school, and college students (Ghosh and Roy, 2017; Ziegler and Opdenakker, 2018; Li et al., 2019), and it produces many adverse effects such as negative emotions, anxiety and depression, lower learning efficiency, lower academic self-esteem, and academic pressure (Romano, 1996; Klassen et al., 2010; Klibert et al., 2016; Krispenz et al., 2019). Previous studies have investigated middle-school and college students, but postgraduates have rarely been observed comparatively. The number of students pursuing master’s degrees has increased worldwide, and unlike younger students, graduate students usually receive little supervision from teachers and parents. Postgraduates therefore may have more opportunity to procrastinate; in fact, it has been demonstrated that more than 70% of graduate students procrastinate academically (Hu, 2008). In light of this, we explored the influencing mechanism of academic procrastination in postgraduates in order to provide intervention or guidance for such students.

### Academic Self-Efficacy and Academic Procrastination

Early procrastination theories, such as the expected value theory (Wigfield and Eccles, 2000), the Temporal Motivation Theory (TMT) (Steel and Konig, 2006), and the time-oriented two-dimensional model (Strunk et al., 2013), attached importance to the effect of self-efficacy as a motivational factor on procrastination behavior. Zhang and Feng (2020) put forward the Temporal Decision Model (TDM), focusing on the microscopic process of procrastination behavior: delayed decision-making. According to the model, the core of procrastination is the “do-procrastinate” or “do-not-procrastinate” decision-making process. When the negative process experience of performing a task is greater than the expected outcome utility at present, then the individual will choose to delay the task. The motivation to engage in a task comes from the result of the task, and the motivation to delay comes from the negative cognition of the process involved.

According to Bandura, self-efficacy refers to individuals’ beliefs about whether they can finish a certain task. Academic self-efficacy is a special category of self-efficacy and refers to learners’ judgment of their own ability and the action ability of the set learning goals to be implemented and achieved (Bandura, 1977). Self-efficacy can affect behavior through four processes: cognitive, motivational, affective, and selective (Bandura, 1993). On the one hand, high self-efficacy promotes individuals’ positive expectations of task results; on the other hand, it also reduces individuals’ negative experience of the task process, thus inhibiting procrastination. Self-efficacy not only influences or determines people’s choice of behaviors but can also affect people’s persistence and efforts (Bandura, 1977, 1993). Procrastination is manifested as a voluntary delay of the scheduled plan even though we know we should not; this also means that the persistence of individual behaviors is not strong. The influence of academic self-efficacy on academic procrastination has been recognized by many researchers. Meta-analysis also shows that self-efficacy is an important and stable predictor of procrastination (Steel, 2007). The results of other studies on the relationship between academic self-efficacy and academic procrastination are also relatively consistent; that is, academic self-efficacy is significantly negatively correlated with academic procrastination, and academic self-efficacy negatively predicts academic procrastination (Ge et al., 2018; Ziegler and Opdenakker, 2018; Przepiorka et al., 2019). Some researchers have suggested that low self-efficacy to self-regulate predicts higher levels of procrastination (Klassen et al., 2008, 2010). Other studies have shown that academic self-efficacy mediates the effects of other variables on academic procrastination (Zhang et al., 2018). Recently, one study revealed that an academic self-efficacy intervention could reduce academic procrastination (Krispenz et al., 2019). Most studies have focused on the relationship between academic self-efficacy and academic procrastination; rarely has the influence mechanism between the two variables been addressed (Hen and Goroshit, 2014; Kandemir, 2014).

### Academic Self-Efficacy, Self-Control, and Procrastination

The procrastination decision-making model holds that the core process of procrastination is the decision-making process of “doing now or doing in the future” and that self-control is the key factor affecting this decision-making process (Zhang et al., 2019). The influencing factors of procrastination are self-control and utility assessment. Self-control regulates individual behavior in a top-down manner, thus reducing procrastination (Zhang and Feng, 2017). Academic self-control refers to an individual’s ability to adjust learning behavior in order to achieve a goal in academic development, with the individual’s body, mind, behavior, external environment, and events as the objects, and social requirements and self-concept as the standards (Zhang, 2006; Duckworth et al., 2019). Duckworth et al. (2019) pointed out two features of self-control: it is necessarily self-initiated, and, more important, it only occurs when an individual makes a choice between something with long-term significance and something with immediate appeal. However, people tend to choose more concrete tasks that need to be performed immediately and ignore more abstract tasks in the distant future, so they are more likely to procrastinate (Gröpel and Steel, 2008). The strength model of self-control states that a state of loss—that is, when self-control is reduced—affects an individual’s decision-making ability (Baumeister et al., 2007). The strength model of self-control also considers emotion and motivation as important factors affecting the strength of self-control (Baumeister et al., 2007; Boucher and Kofos, 2012). Thus, self-control as a volitional factor may have an inhibitory effect on procrastination behavior. Numerous studies showed that self-control negatively predicted procrastination (Kuhnel et al., 2018; Przepiorka et al., 2019), and trait self-control interacted with sleep quality in impacting next-day work procrastination (van Eerde and Venus, 2018). Studies have also confirmed that academic self-control negatively predicts academic procrastination (Ariely and Wertenbroch, 2002).

On the other hand, people with high academic self-efficacy have a high degree of “persistence and effort” in the implementation of the whole learning plan (Bandura, 1977). This “persistence and effort,” or self-control, means that students can constantly adjust their learning behaviors to complete goals on time and avoid procrastination. Studies have found that academic self-efficacy is positively correlated with academic self-control, and academic self-efficacy can positively predict academic self-control (Ein-Gar and Steinhart, 2017; Chen et al., 2019). Further, academic self-efficacy mediates the influence of other variables on academic self-control (Zhao and Zhang, 2018). Based on previous research, this study established the hypothesis that academic self-control plays a mediating role in the influence of academic self-efficacy on academic procrastination.

### Gender Difference

Previous studies have shown significant gender differences in academic self-efficacy, with male students often showing higher academic self-efficacy (Li, 2010). In a large meta-analysis of 187 studies containing 247 independent studies (*n* = 68,429), Huang (2013) found an overall gender difference in the level of academic self-efficacy, with males having higher self-efficacy. Huang (2013) found gender differences among 15- to 18-year-olds and students 23 or older, and culture appeared to have no moderating effect on gender differences in academic self-efficacy. However, in contrast to self-efficacy, self-control in girls is significantly higher than that in boys (Kremen and Block, 1998; Shoenberger and Rocheleau, 2017; Hamama and Hamama-Raz, 2019; Zavala et al., 2019). Therefore, we speculated that the influence of academic self-efficacy on academic self-control may be significantly different depending on gender, and the mediating effect of academic self-control may also be different according to gender.

Some studies showed a significant difference in the degree of academic procrastination between genders (Ozer et al., 2009; Ghosh and Roy, 2017) and some did not (Klibert et al., 2011, 2016). Ozer et al. (2009) pointed out gender differences in the causes of procrastination; significantly more female students than male students reported greater academic procrastination because of laziness and fear of failure, while more male students than female students reported more academic procrastination as a result of risk taking and rebellion against control. Ge et al. (2018) found that gender played a moderating effect in the influence of academic self-efficacy on academic procrastination in junior high school students. Specifically, the academic self-efficacy of male students could significantly predict their academic procrastination behavior, which was not true for female students. For that reason, this study also examined whether gender played a moderating role in the influence of academic self-efficacy on academic procrastination.

### The Present Study

**FIGURE 1:**
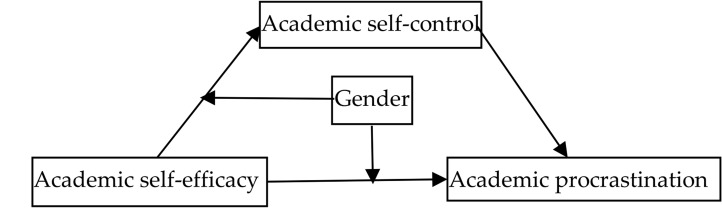
Hypothetical models of academic self-efficacy, academic self-control, academic procrastination, and gender.

Most previous studies discussed the motivational (academic self-efficacy) and volitional (academic self-control) causes of procrastination, specifically the relationship between academic self-efficacy, academic self-control, and academic procrastination, as well as gender differences. There is a scarcity of comprehensive research on the interaction mechanism between these variables, however, especially for graduate students, who work more independently than younger students. This study investigated the mediating effect of academic self-control between academic self-efficacy and academic procrastination. Based on the significant gender differences in academic self-efficacy, self-control, and procrastination found in previous studies, we investigated whether gender variables could moderate the influence of academic self-efficacy on academic self-control and the influence of academic self-efficacy on academic self-control. We created four research hypotheses (Figure 1):

(1) Academic self-efficacy has a negative and significant impact on academic procrastination.
(2) Academic self-efficacy affects academic procrastination through academic self-control.
(3) Gender variables play a moderating role in the direct effect of academic self-efficacy on academic procrastination.
(4) Gender variables can moderate the influence of academic self-efficacy on academic self-control, thus significantly moderating the mediating effect of academic self-control.

## Materials and Methods

### Participants

We used a random cluster sampling of full-time graduate students in a university town of a Chinese city. Professors distributed 650 questionnaires in classes, and participants’ informed consent was obtained before the 577 valid questionnaires were collected. The effective recovery rate was 88.8%. Among them, 222 were in their first year, 188 in their second year, and 167 in their third year; 351 were women, and 226 were men. The average age was 23.93 ± 1.67 years.

### Measures

#### Questionnaire on Academic Self-Efficacy of Postgraduate Students

Li (2010) divided academic self-efficacy of postgraduates into three parts: the sense of self-efficacy of course learning (e.g., “I have strong ability to study independently in the professional course of study”); the sense of self-efficacy of scientific research ability (e.g., “My scientific research ability is relatively strong”); and the sense of self-efficacy of social practice ability (e.g., “I can apply my knowledge and skills to social practice”). Based on this division, Li (2010) developed a scale of academic self-efficacy of postgraduates. This questionnaire includes 18 questions and adopted a Likert 5-level scoring scale: “strongly disagree,” “disagree,” “uncertain,” “agree,” and “strongly agree.” There was no reverse scoring on the scale. The higher the score, the higher the sense of self-efficacy. In this study, Cronbach’s α was 0.87 and the structural validity index was good (χ^2^*/df* = 2.33, CFI = 0.95, TLI = 0.91, SRMR = 0.06, RMSEA = 0.05).

#### Academic Self-Control Questionnaire

The scale used in this study was revised by Zhang (2006). The revised questionnaire on academic self-control has a total of 38 questions on a 5-point scale including three dimensions: a sense of self-control (e.g., “I can apply my knowledge and skills to social practice”); self-control tendency (e.g., “I always think positively in class”); and self-control strategy (e.g., “There are both long plans and short arrangements in my learning”). The higher the score, the higher the ability of academic self-control. In this study, Cronbach’s α was 0.82, and the structural validity of the questionnaire was good (χ^2^*/df* = 4.46, CFI = 0.88, TLI = 0.91, SRMR = 0.07, RMSEA = 0.07). A multivariate Lagrange multiplier test identified modifications to improve the model fit. The modified index showed the highest MI between item 3 and item 13. Because there are enough items (12) in this dimension (a sense of self-control), we deleted the two items according to some researchers’ advise (Anderson and Gerbing, 1988; Landis et al., 2009), ran again the CFA after deleting, and found indicators better fitting (χ^2^*/df* = 3.04, CFI = 0.91, TLI = 0.93, SRMR = 0.05, RMSEA = 0.05), so 36 items were retained in the final questionnaire.

#### Questionnaire for Academic Procrastination of Postgraduate Students

We used the study procrastination questionnaire for Chinese master’s students compiled by Hu (2008). The original questionnaire consists of two parts. The first part examines the procrastination of master students in six academic activities (writing term papers/assignments, publishing papers/research reports, reading literature, tutoring assignments, participation assignments, thesis proposal/graduation thesis) and three problems: the tendency to procrastinate (“Have you delayed this task?”); problems caused by procrastination (“Did delaying this task cause any trouble?”); and expectations of lower procrastination (“Do you want to reduce procrastination in this task?”). The second part examines the reasons for procrastination. Since this study only investigated the procrastination tendency of postgraduate students, we used only the first question in the first part of this scale. Participants were asked to circle the option that best reflected their actual procrastination tendency on a 5-point Likert scale. The higher the score on the first item of each academic activity (i.e., questions 1, 4, 7, 10, 13, and 16), the more serious the tendency to delay. In this study, Cronbach’s α was 0.85 and the structural validity index was good (χ^2^*/df* = 2.32, CFI = 0.91, TLI = 0.92, SRMR = 0.07, RMSEA = 0.05).

### Statistical Processing

SPSS 24.0 (Corporation, 2015) was used to conduct a common method bias test, a gender difference test of main variables, correlation analysis, and scale reliability analysis on the data. Mplus 8.1 (Muthén and Muthén, 2017) was used to analyze the validity of the questionnaires’ structure and to verify the hypothetical model with LMS (latent moderate structural equations) bootstrapping procedures (Preacher and Hayes, 2004). Model fit indices generally used to interpret the fit of structural equation models, such as CFI, TLI, RMSEA, and χ^2^, have not been developed for LMS models. Alternatively, by referring to Maslowsky et al. (2015), we first examined the fitting degree [Critical value: χ^2^/*df*<5, CFI > 0.9, TLI > 0.9, SRMR < 0.08, RMSEA < 0.08 (Kline, 2010)] of simple mediation model (Model 0, where the interaction is not estimated). And then to test the moderated mediation model (Model 1, where the interaction is estimated), there are two ways to determine whether the latter fit is better: The first is to use AIC and BIC judgment. If AIC and BIC become smaller or remain unchanged, then the model is improved or at least not deteriorated because the bigger AIC and BIC are, the more information is lost (Sardeshmukh and Vandenberg, 2017). The second method is using the log-likelihood ratio test, where the relative fit of Model 0 and Model 1 is compared. The value of −2LL (log-likelihood for Model 0 – log-likelihood for Model 1) was calculated according to the H_0_ value in the Mplus results. The −2LL value was approximately subject to chi-square distribution. If the chi-square test of the −2LL is significant, it means that Model 1 is better (Maslowsky et al., 2015).

## Results

### Common Method Bias Test

The Harman single factor test was conducted on all the measured items in this study, and there were 18 factors whose eigenvalue was >1. The first factor accounted for 19.53% of the total variation, <40% of the critical value, indicating that there was no serious common method bias in this study (Zhou and Long, 2004). Confirmatory factor analysis was performed using Mplus 8.1 (Muthén and Muthén, 2017), and the single factor model fit was extremely poor (χ^2^*/df* = 42.36, CFI = 0.26, TLI = 0.37, RMSEA = 0.15), indicating that the common method bias in this study was not significant (Xiong et al., 2012).

### Gender Differences and Correlation Among Variables

The gender difference test and correlation among variables of academic self-efficacy, academic self-control, and academic procrastination of master’s students are shown in Table 1. In this study, only academic self-efficacy had significant difference in relation to gender; specifically, the academic self-efficacy of male students was significantly higher than that of female students. In addition, academic self-efficacy was positively correlated with academic self-control and negatively correlated with academic procrastination. Academic self-control was negatively correlated with academic procrastination.

**TABLE 1:** Gender difference test and correlation of main variables.

**Variables**	**Male *M*±*SD***	**Female *M*±*SD***	***T***	**1**	**2**	**3**
1. Academic self-efficacy	3.76 ± 0.55	3.54 ± 0.56	3.08**	1		
2. Academic self-control	3.32 ± 0.40	3.24 ± 0.47	1.47	0.53**	1	
3. Academic procrastination	2.65 ± 0.82	2.67 ± 0.78	−0.14	−0.31**	−0.42**	1
4. Skewness				0.01	−0.36	0.20
5. Kurtosis				−0.07	0.87	−0.30

**P < 0.01.

The skewness and kurtosis coefficient of academic self-efficacy, academic self-control, and academic procrastination are shown in Table 1. The absolute value is <1, indicating that the data are approximate to normal distribution. Therefore, maximum likelihood (ML) can be used (Muthén and Muthén, 2017).

### The Mediating Effect of Academic Self-Control and the Moderating Effect of Gender

According to the LMS procedure recommended by Maslowsky et al. (2015), a moderated mediating model was established to test the direct effect of academic self-efficacy on academic procrastination and whether the mediating effect of academic self-control between academic self-efficacy and academic procrastination was moderated by gender. First, we tested the simple mediation model (Model 0, where the interaction is not estimated; gender variables, which were converted to dummy variables before this operation). The model test results showed that all fitting indices of the Model 0 reached the critical level (χ^2^*/df* = 1.34, CFI = 0.99, TLI = 0.98, RMSEA = 0.004, SRMR = 0.04). Then, the moderated mediation model (Model 1, where the interaction is estimated) was examined. The test results showed that the AIC value of Model 1 was 7.87 lower than that of Model 0 (Model 0: AIC = 6113.99; Model 1: AIC = 6106.12), and the BIC value decreased by 0.69 (Model 0: BIC = 6261.54; Model 1: BIC = 6260.85), which indicates that Model 1 was a better fit than Model 0 (Sardeshmukh and Vandenberg, 2017). In addition, Log Likelihood of Model 1 with latent regulation was -3001.06. Compared with Model 0, Log Likelihood = -3016.00, its value increased by 15.94; that is, the −2LL value was 15.94. The degree of freedom increased by 1, and the chi-square test of the −2LL was significant (*p* < 0.01); thus, Model 1 had better fit (Maslowsky et al., 2015) and the test of Model 1 could be performed. The third step was to test the moderated mediation model with latent regulation.

**FIGURE 2:**
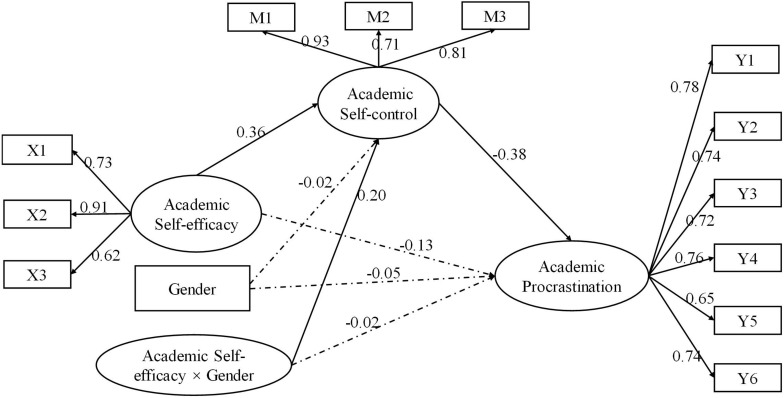
Chart of academic self-efficacy, academic self-control, and gender influence on academic procrastination (standardized). Note: *n* = 577. The solid line means significant in the 95% confidence interval, and the dashed line means insignificant in the 95% confidence interval. To keep the graph clean, residuals are unmarked.

**FIGURE 3:**
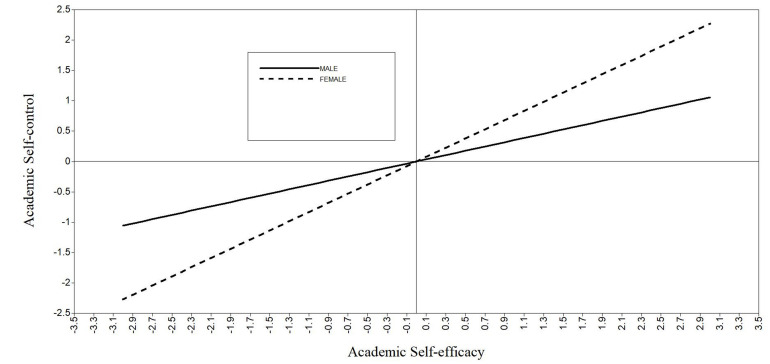
The moderating effect of gender on academic self-efficacy and academic self-control.

The results (see Figure 2) showed that (1) academic self-efficacy had significant predictive effect on academic self-control (β = 0.36, *p* < 0.001), while gender had no significant predictive effect on academic self-control (β = −0.02, *p* > 0.05). The interaction between academic self-efficacy and gender had a significant predictive effect on academic self-control (β = 0.20, *p* < 0.001). (2) The direct prediction effect of academic self-efficacy on academic procrastination was not significant (β = −0.13, *p* > 0.05), the effect of gender on academic procrastination was not significant (β = −0.05, *p* > 0.05), and the interaction between gender and academic self-efficacy was not significant in predicting academic procrastination (β = −0.02, *p* > 0.05), but the predictive effect of academic self-control on academic procrastination was significant (β = −0.38, *p* < 0.001). (3) The mediating effect was significantly different between male and female groups (β = −0.29, 95% CI = [−0.61, −0.10]), and the mediating effect of academic self-control was significant in both male and female groups. To be specific, the mediating effect of academic self-control in female students (β = −0.54, 95% CI = [−0.94, −0.24]) was significantly stronger than that in male students (β = −0.25, 95% CI = [−0.56, −0.08]). (4) The moderating effect of gender on academic self-efficacy and academic self-control was further analyzed by simple slope test. The results showed that compared with male students, female students’ academic self-efficacy had a more significant predictive effect on academic self-control; Simple slope (female) = 0.75, *p* < 0.001, Simple slope (male) = 0.35, *p* < 0.001 (Figure 3). That is, in terms of the effect of academic self-efficacy on academic self-control, with the increase of academic self-efficacy, both male and female graduate students had a significant increase in academic self-control. Compared with male students, female students had a larger increase.

In conclusion, we proved that academic self-control played a completely mediating effect between academic self-efficacy and academic procrastination. The mediating role of academic self-control between academic self-efficacy and academic procrastination was moderated by gender variables, and the moderating role occurred in the first half of the path.

## Discussion

### The Mediating Effect of Academic Self-Control

This study found that academic self-control played a completely mediating role between academic self-efficacy and academic procrastination, which revealed the influence of academic self-efficacy on academic procrastination. The results can be explained from several theoretical perspectives. First, Bandura’s self-efficacy theory states that self-efficacy affects individual behavioral activities through four action mechanisms (Bandura, 1993). Second, according to the strength model of self-control, individual motivation and emotion are the important factors influencing self-control when facing tasks, and self-control affects the decision-making process and effectiveness (Baumeister et al., 2007). Third, the procrastination decision-making model demonstrates that self-control is the core factor affecting the process of procrastination decision-making (Zhang and Feng, 2020). This study combines the above theoretical models. In terms of action mechanisms, individuals with high academic self-efficacy when facing a task will set high goals (cognitive process), have stronger motivation (motivation process), and have fine emotions (emotional process). High self-efficacy is beneficial to the individual’s good academic self-control and final selection (selection process), completion of the task goal, and avoidance of academic procrastination. However, individuals with low academic self-efficacy cannot maintain strong motivation (motivation process) even if they set low task goals (cognitive process) when facing task situations. They may also suffer from anxiety and depression (emotional process) and lower self-control of tasks, and they are more likely to choose avoidance, procrastination, and other self-defeating behaviors.

Self-control, as the ante-dependent variable of individual behaviors, is also reviewed in other studies on topics such as mobile phone addiction tendency (Han et al., 2017), aggression (Neaverson et al., 2020), and many other behaviors (Louderback and Antonaccio, 2020). Self-control has the most direct decisive effect on individual behavior (Zimmerman and Risemberg, 1997), as supported by our study.

In addition, one dimension of the self-control scale used in this study, “sense of self-control,” means that the self-efficacy for self-control is similar to the “self-efficacy for self-regulation” (Klassen et al., 2008) to some extent. Klassen et al. (2008) found that self-efficacy for self-regulation was most predictive of procrastination tendencies among certain self-variables (others were self-regulation, academic self-efficacy, and self-esteem). Self-regulated learning embraces not only the volitional process of self-control but also motivational processes and learning strategies (Duckworth et al., 2019). The results of our study are partly consistent with those of Klassen et al. (2008, 2010).

### The Moderating Effect of Gender

In this study, the moderating effect of gender was reflected in the mediating effect of academic self-control between academic self-efficacy and academic procrastination, which was embodied in the influence of academic self-efficacy on academic self-control (the first half of the path). To be specific, academic self-control played a significant mediating role in both male and female groups, but female postgraduate students were stronger than male postgraduate students in the influence of academic self-efficacy on academic self-control and the mediating role of academic self-control. According to our data analysis, this difference between male and female groups was mainly caused by the significant difference of academic self-efficacy between male and female groups. The academic self-efficacy of male students was significantly higher than that of female students, which is consistent with the conclusions of most studies (Huang, 2013). That is, men tended to show more confidence in most aspects, but this high confidence did not lead to greater academic self-control or a significant reduction in academic procrastination.

On the other hand, some studies found that groups of females performed better on self-control (Kremen and Block, 1998; Zavala et al., 2019). In addition, studies also found that the mediating effect of self-control in female students was significantly higher than that in male students (Luo et al., 2018). However, in this study, there was no gender difference in academic self-control. This may be because “self-control” refers to emotion or short-term behaviors in which boys and men are more impulsive, easily excited physically, and unable to restrain themselves (Luo et al., 2018). However, learning is a long-term behavior, and males’ impulsivity and excitability are not obvious in long-term behavior, so there is no significant difference in the performance of academic self-control between men and women. Another possible reason is that our participants were older. In previous studies on self-control gender differences, participants were all primary school students, middle school students (Hamama and Hamama-Raz, 2019), and college students (Duckworth et al., 2019). Our male postgraduate students were more mature and knowledgeable about the meaning of learning and self-control, which may explain the differences between females and males here. This should be verified in future studies. However, the results of our study indicate that the direct effect of academic self-efficacy on academic procrastination was not significant, and the moderating effect of gender was also not significant. In other words, the influence of academic self-efficacy on academic procrastination was completely mediated by academic self-control for both men and women.

In sum, although the academic self-efficacy of male students was significantly higher than that of female students, the effect of academic self-efficacy on academic self-control was significantly lower than that of female students. The mechanism of academic self-efficacy affecting academic procrastination via academic self-control was significantly different between male and female postgraduate students.

## Conclusion

The measurements of variables in this study were cross-sectional self-reports. Follow-up and experimental methods should be considered to make the measurements of variables more objective and accurate.

It should be pointed out that Bandura’s self-efficacy theory and the strength model of self-control only involve “general self-efficacy” and “general self-control theory,” and this study only proves the applicability of these two theories in the specific behavior of “learning.” In addition, because this study investigated several variables (academic self-efficacy, academic self-control, and academic procrastination), all belonging to special psychological traits, the process of motivation, the emotion process, and the selection process mentioned in the self-control strength model were not investigated in this study. The effect of situational self-efficacy on situational procrastination should be examined in future studies.

On the other hand, compared with other student groups, the relationship between postgraduate students’ academic procrastination, self-efficacy, and self-control was more explicit and objective, because postgraduate students were less affected by external variables and their learning was more independent. For example, the academic procrastination behavior of middle school students was related to perceived support from teachers (Mouratidis et al., 2018). In the future, the academic procrastination behavior of different groups can be compared and studied to explore the differences in the influencing mechanisms.

As indicated in this study, to reduce the procrastination behavior of postgraduate students, we can enhance their sense of academic self-efficacy and cultivate academic self-control, keeping in mind that improving men’s sense of academic self-efficacy will be less effective than it is for women. For male postgraduate students, more effort should be made to reduce their academic procrastination by directly cultivating strategies for and methods of academic self-control.

## Data Availability Statement

The datasets generated for this study are available on request to the corresponding author.

## Ethics Statement

The studies involving human participants were reviewed and approved by the Research Ethics Committee in the School of Psychology in Guizhou Normal University. The patients/participants provided their written informed consent to participate in this study.

## Author Contributions

SZ conceived the experimental design. GL contributed to the data collection, preparation of the statistical analyses, and drafted the manuscript. JH was mainly responsible for the language polishing and proofreading. GC and YP were dedicated to refining the manuscript. All authors have read and approved the final draft.

## Conflict of Interest

The authors declare that the research was conducted in the absence of any commercial or financial relationships that could be construed as a potential conflict of interest.
