# Supplementary material for: Academic Self-Efficacy and Postgraduate Procrastination: A Moderated Mediation Model
Source: Front Psychol. 2020 Jul 24;11:1752. doi: 10.3389/fpsyg.2020.01752 (PMC7393210; doi:10.3389/fpsyg.2020.01752)
Supplement: Supplementary file 1 [file Table_1.docx]

Supplementary Material

**Note:** The questionnaire in study is in Chinese, here it is translated into English for review.

***Questionnaires***

Dear students,

This questionnaire consists of three scales. Please read the "instruction" carefully before answering each part of the questionnaire. There is no definite right or wrong answer when filling the questionnaire, because everybody has his or her own unique growth background and feelings, so please read the questions carefully and choose as soon as possible according to your current real reaction and perception, do not consider it too long, tick the number that fits your situation best. **Please fill in all the questions. You can only choose one answer for each question, do not choose more or omit.**

This survey is conducted anonymously. Your answers will only be used for this research. Your answers will be kept strictly confidential.

**Thank you for your support!**

Basic information:

Grade: ; Occupation: ; Gender: ; Age years old.

***Scale 1 (18 questions in total)***

Please tick the following 18 statements

Very disagree (1) ; Disagree (2); Uncertain (3); Agree (4); Very agree (5).

1. My scientific research ability is relatively strong. 1, 2, 3, 4, 5

2. I am capable of publishing papers of certain academic value. 1, 2, 3, 4, 5

3. I can apply my knowledge and skills to scientific research activities. 1, 2, 3, 4 ,5

4. I am capable of excellently completing the scientific research tasks assigned by my tutor. 1, 2, 3, 4, 5

5. I like to challenge the difficulties in the study of my major problem. 1, 2, 3, 4, 5

6. I can keenly capture the hot issues in my discipline and come up with novel ideas. 1, 2, 3, 4, 5

7. I actively seek out research issues and research fields that I am interested in and conduct researches. 1, 2, 3, 4,5

8. When engaged in scientific research activities, I can communicate and cooperate well with others. 1, 2, 3, 4, 5

9. When confronted with difficulties in scientific research activities, I still believe in my scientific research ability. 1, 2, 3, 4, 5

10. I have strong ability to study independently in the professional course study. 1, 2, 3, 4, 5

11. I can excellently finish the course tasks assigned by the teacher of specialized courses. 1, 2, 3, 4, 5

12. In my major, I can put forward some valuable ideas. 1, 2, 3, 4, 5

13. I have the ability to thoroughly understand the classics of my major. 1, 2, 3, 4, 5

14. In specialized courses study, I can interact well with my teachers. 1, 2, 3, 4, 5

15. In social practice, I can communicate and cooperate well with others. 1, 2, 3, 4, 5

16. When carrying out social practice activities, I can achieve the predetermined goals well. 1, 2, 3, 4, 5

17. I can apply my knowledge and skills to social practice. 1, 2, 3, 4, 5

18. I have strong ability in social practice (internship or assistant management). 1, 2, 3, 4, 5

***Scale 2 (18 questions in total)***

Instructions: please evaluate the extent to which you are delaying or procrastinating in the following learning activities, and tick "√" on the corresponding choices. Please do not omit or choose more than one choice.

Note: if in "Are you procrastinating on this task?" Choose 1. Never procrastinate. In "did the procrastination in this task cause you trouble?" It's never a problem to choose 1 from 1.

**I Write term paper assignments.**

1. ***Have you delayed this task?***

***1 never 2 rarely 3 occasionally 4 often 5 always***

2. Did the delaying in this task cause you any trouble?

*1 never 2 rarely 3 occasionally 4 often 5 always*

3. Do you want to reduce procrastination in this task?

*1 never 2 rarely 3 occasionally 4 often 5 always*

**Ⅱ Papers published research report**

1. ***Have you delayed this task?***

***1 never 2 rarely 3 occasionally 4 often 5 always***

5. Did the delaying in this task cause you any trouble?

*1 never 2 rarely 3 occasionally 4 often 5 always*

6. Do you want to reduce procrastination in this task?

*1 never 2 rarely 3 occasionally 4 often 5 always*

**Ⅲ Read academic literature**

1. ***Have you delayed this task?***

***1 never 2 rarely 3 occasionally 4 often 5 always***

8. Did the delaying in this task cause you any trouble?

*1 never 2 rarely 3 occasionally 4 often 5 always*

9.Do you want to reduce procrastination in this task?

*1 never 2 rarely 3 occasionally 4 often 5 always*

**Ⅳ Mentor assignments**

1. ***Have you delayed this task?***

***1 never 2 rarely 3 occasionally 4 often 5 always***

11. Did the delaying in this task cause you any trouble?

*1 never 2 rarely 3 occasionally 4 often 5 always*

12.Do you want to reduce procrastination in this task?

*1 never 2 rarely 3 occasionally 4 often 5 always*

**V Participating tasks: meeting with tutors, participating in group academic activities, academic lectures, etc**

1. ***Have you delayed this task?***

***1 never 2 rarely 3 occasionally 4 often 5 always***

14. Did the delaying in this task cause you any trouble?

*1 never 2 rarely 3 occasionally 4 often 5 always*

1. Coincidence. Do you want to reduce procrastination in this task?

*1 never 2 rarely 3 occasionally 4 often 5 always*

**Ⅵ Thesis opening/writing graduation thesis**

1. ***Have you delayed this task?***

***1 never 2 rarely 3 occasionally 4 often 5 always***

1. Did the delaying in this task cause you any trouble?

*1 never 2 rarely 3 occasionally 4 often 5 always*

1. Do you want to reduce procrastination in this task?

*1 never 2 rarely 3 occasionally 4 often 5 always*

***Scale 3 (38 questions in total)***

Guide language: please evaluate the consistency of each statement according to the actual situation of your study, 1 is very inconsistent, 5 is very consistent, tick "√" on the options that match with you.

Very inconsistent → very consistent

1. I can always spare a reasonable time to rest in study so as to maintain full energy. [1, 2, 3, 4, 5]

2. I will try to concentrate when something distracts me. [1, 2, 3, 4, 5]

3. Without other people's supervision, I can hardly concentrate on my study. [1, 2, 3, 4, 5]

4. I will set learning goals for myself when I study. [1, 2, 3, 4, 5]

5. I don't want to listen to my teacher when he or she doesn't lecture well in class. [1, 2, 3, 4, 5]

6. I can always give up the activities I am interested in for the sake of study. [1, 2, 3, 4, 5]

7. I will review regularly. [1, 2, 3, 4, 5]

8. I listen in when others chat while studying. [1, 2, 3, 4, 5]

9. When I meet problems I don't understand, I will figure it out in time. [1, 2, 3, 4, 5]

10. When meeting difficulties in study, I will ask others. [1, 2, 3, 4, 5]

11. I also know to study hard, but I just can't control myself. [1, 2, 3, 4, 5]

12. I can always achieve my learning goals. [1, 2, 3, 4, 5]

13. I always make the most of my time. [1, 2, 3, 4, 5]

14. There are both long plans and short arrangements in my learning. [1, 2, 3, 4, 5]

15. I can always concentrate for a long time when I study. [1, 2, 3, 4, 5]

16. When the conflict between favorite activities and learning, I always go to the activity. [1, 2, 3, 4, 5]

17. I often summarize the learning experience and methods. [1, 2, 3, 4, 5]

18. My bad mood often affect my study. [1, 2, 3, 4, 5]

19. While studying, other people are talking about the topic I am interested in, I will join in. [1, 2, 3, 4, 5]

20. The unfinished learning tasks (such as homework, etc.) will be timely completed. [1, 2, 3, 4, 5]

21. I will not ask others when I can not solve the learning problems. [1, 2, 3, 4, 5]

22. I can always concentrate in class. [1, 2, 3, 4, 5]

23. I always put off what can be done today till tomorrow. [1, 2, 3, 4, 5]

24. I always think positively in class. [1, 2, 3, 4, 5]

25. I usually don't review what I've learned. [1, 2, 3, 4, 5]

26. I will arrange enough time to review before the exam. [1, 2, 3, 4, 5]

27. I don't work hard on subjects that aren't important. [1, 2, 3, 4, 5]

28. I will find relevant scientific books for myself. [1, 2, 3, 4, 5]

29. I always test my plans against the achievement of my goals. [1, 2, 3, 4, 5]

30. I can always try to forget the unpleasant mood, and re-involve into my study. [1, 2, 3, 4, 5]

31. I can keep on studying with all the noise around me. [1, 2, 3, 4, 5]

32. I will take the initiative to correct mistakes in homework or exams. [1, 2, 3, 4, 5]

33. I can always make good use of spare time to study. [1, 2, 3, 4, 5]

34. I will take the initiative to answer the teacher's questions in class. [1, 2, 3, 4, 5]

35. Study plan is too rigid, I usually do not make. [1, 2, 3, 4, 5]

36. When I come across a problem in study, I always want to delve into the result. [1, 2, 3, 4, 5]

37. I always take notes in class. [1, 2, 3, 4, 5]
